# Regulation of Social Stress and Neural Degeneration by Activity-Regulated Genes and Epigenetic Mechanisms in Dopaminergic Neurons

**DOI:** 10.1007/s12035-020-02037-7

**Published:** 2020-08-03

**Authors:** Clement Kent, Pavan Agrawal

**Affiliations:** 1 Department of Biology, York University, Toronto, ON M3J 1P3 Canada; 2 Centre for Molecular Neurosciences, Kasturba Medical College, Manipal Academy of Higher Education, Manipal, Karnataka 576104 India

**Keywords:** Dopamine, Activity-regulated genes, Immediate early genes, Parkinson’s disease, Social isolation, *Drosophila melanogaster*

## Abstract

Transcriptional and epigenetic regulation of both dopaminergic neurons and their accompanying glial cells is of great interest in the search for therapies for neurodegenerative disorders such as Parkinson’s disease (PD). In this review, we collate transcriptional and epigenetic changes identified in adult *Drosophila melanogaster* dopaminergic neurons in response to either prolonged social deprivation or social enrichment, and compare them with changes identified in mammalian dopaminergic neurons during normal development, stress, injury, and neurodegeneration. Surprisingly, a small set of activity-regulated genes (ARG) encoding transcription factors, and a specific pattern of epigenetic marks on gene promoters, are conserved in dopaminergic neurons over the long evolutionary period between mammals and insects. In addition to their classical function as immediate early genes to mark acute neuronal activity, these ARG transcription factors are repurposed in both insects and mammals to respond to chronic perturbations such as social enrichment, social stress, nerve injury, and neurodegeneration. We suggest that these ARG transcription factors and epigenetic marks may represent important targets for future therapeutic intervention strategies in various neurodegenerative disorders including PD.

## Introduction

Neurons and glia can enter into, stay in, and exit from epigenetically regulated semi-stable states. Cell fate choice during normal neuronal development is a one-way journey through pro-neural states to a stable, differentiated mature neuron. However, after injury, several states may be maintained for long periods, possibly followed by a reversal to a normal mature neural state [1]. Not all neural state changes are related to disease, injury, or development; for example, changes in environmental conditions and exposure to stressors may cause groups of neurons to become more or less active for prolonged periods. A well-studied example of this is the effect of prenatal and early life stress, which produces epigenetic modifications in neural genes resulting in persistent behavioral changes [2–4].

We studied epigenetic and transcriptional changes in dopaminergic neurons (DANs) in the fruit fly *Drosophila melanogaster*, where prolonged social isolation and social enrichment cause restructuring of the epigenetic landscape [5]. DANs are involved in neuronal differentiation [6] and the response to stress in mammals [7, 8] but their influence in these conditions is less studied in insects. Such long-lasting but ultimately reversible state changes in *Drosophila* usually involve various neuropeptides [9, 10] and neurotransmitters including dopamine [5, 11, 12]. Prolonged social isolation in *Drosophila* can drastically affect behavior as in mammals; for instance, socially isolated *Drosophila* show a reduction in sleep [13, 14] and an increase in aggression [15, 16], both modulated by DAN signaling.

We found that in *Drosophila*, changes in social experience produce global changes in the epigenetic landscape of the DAN network. We also found that *Drosophila* activity-regulated genes (ARGs) [17] are upregulated in DANs for several days following social enrichment [5]. ARGs are genes whose transcription is regulated by neural activity or other stimuli [17] and a subset of these is also known as immediate early genes (IEGs) in vertebrates [18, 19], whose activation in neurons after learning has been utilized to identify brain regions involved in learning, stress, and neuronal plasticity [17, 20–22]. We focus on four ARGs significantly upregulated in fly DANS by social experience [5] whose homologs in mammals are CREB, EGR, NR4A1, and KLF11 (Table 1). Unlike classical IEG expression, which increases rapidly after stimulation and decreases in hours, the sustained increase in these four ARGs is reminiscent of the multi-day increase in c-Jun found after both sciatic nerve sectioning [45] and axotomy of the dopaminergic nigrostriatal pathway [46]. In addition, c-Jun is essential in Schwann cells for them to respond appropriately to injury [47]. In this review, we compare and contrast state changes in mature adult neurons resulting from injury with those resulting from other stimuli, with a particular focus on whether ARGs upregulated in mature neurons may use known epigenetic pathways.

**Table 1:** Activity-regulated genes (ARGs) in *Drosophila* and their homologs in mammals. The table summarizes fly ARG transcription factors and their human IEG homologs. These ARGs were upregulated upon social enrichment in *Drosophila* DANs, and their targeted knockdown significantly reduced the extent of sleep modulation by social experience [5]. Representative studies in mammals highlight the role of individual ARGs during neural development, stress, and neurodegeneration and regeneration. For details, please see associated main text

*Drosophila* ARGs	Mammalian ARGs	Key neuronal functions	References
*CrebA*	CREB3L1/OASIS	Downregulated in the brains of socially isolated flies and mice; involved in neuronal regeneration; astrocyte formation; secretory pathway regulation in the ER with roles in PD; upregulated upon prolonged L-DOPA treatment in PD; nuclear localization of HDAC4 reduces CREB signaling and promotes DAN loss.	[23–32]
*Hr38*	Nurr1/NR4A2	Production of DANs from iPSCs; maintenance of adult DANs; reduced *Nurr1* expression in PD; neurotransmitter switching of glutamatergic VTA neurons; upregulation upon L-DOPA administration in PD models.	[33–37]
*Cabut*	KLF11-10	Increased upon social defeat in the prefrontal cortex; regulation of DRD2 dopamine receptor transcription; interaction with epigenetic repressive complexes such as SIN3A, HP1, and with WD40 containing proteins.	[38–40]
*Stripe*	EGR1-4	Schwann cell myelination; interaction with c-Jun; upregulation in birds upon conspecific song; LSD1 interaction and upregulation in high-anxiety phenotypes; maintenance of adult DANs; upregulation upon L-DOPA administration in the PD model.	[37, 41–44]

Here we compare both the epigenetic changes that we found in flies and the role of ARG transcription factors (ARG-TFs) in inducing/blocking behavioral changes, to the roles of ARG and epigenetic changes found in mammalian nerves after injury or during regeneration. First, we discuss the four ARGs that we found to be functionally involved in encoding effects of social experiences in DANs and compare them to their homologous genes in mammals. Second, we briefly review the role of epigenetics in neural transcriptional state regulation. Third, we propose a model to suggest how subsets of neuronal genes may be shifted into new transcriptional/epigenetic states by the role of ARGs in transducing what has been called the “genomic action potential” or gAP [41]. Finally, we speculate how these broadly expressed ARGs can achieve cell-type-specific regulatory effects by interacting with pre-existing transcriptional platforms.

## Four ARG-TFs and Their Homologs

Four ARG-TFs—*CrebA*, *Hr38*, *Cabut*, and *stripe*—show significantly increased transcription in DANs in socially enriched (group housed) flies while others did not [5]. To determine whether these ARGs were functionally involved in the response to social conditions, we reduced the expression of these ARGs by expressing an RNAi construct in subsets of DANs using a tyrosine hydroxylase (TH) promoter-specific driver line—*TH*-GAL4 [48]. This reduced the extent of sleep modulation by social experience [5], suggesting that the ARGs are required in DANs to transduce social inputs into behavioral output. Mammalian homologs of these ARGs serve important functions during neuronal development, injury, and regeneration (Table 1) [47].

### *CrebA*/CREB3L1/OASIS

The regeneration literature is rich in examples of the interplay between ARGs, histone modifications, and neural states, as reviewed by [23], where the authors summarize the roles of histone acetyltransferases (HATs) and histone deacetylases (HDACs) in regeneration and their interplay with cAMP/PKA/CREB signaling. cAMP/CREB signaling regulates neuronal survival and differentiation [49, 50] and confers neuroprotection upon stress [51–53]. Interestingly, CREB1 was shown to be downregulated in the nucleus accumbens shell of socially isolated rats and regulate anxiety-like behaviors [24]. Similar to its role in social isolation and sleep in rodents [24, 54], the CREB family transcription factor CrebA is downregulated in DANs of socially isolated flies [5]. Furthermore, the targeted reduction of CrebA in DANs produced the strongest reduction in sleep phenotype modulation by social enrichment in our study.

CrebA has the closest similarity to CREB3L1/OASIS and CREB3L2/BBF2H7. OASIS (old astrocyte specifically induced substance) was first identified by its overexpression in cultured astrocytes in response to age and in the cerebral cortex in response to injury [25]. It was shown that the distribution of OASIS-positive cells in the cryo-injured cortex was similar to that of glial fibrillary acidic protein (GFAP)-positive cells [25]. OASIS is upregulated in reactive astrocytes after neuronal degeneration induced by kainic acid [26]. OASIS expression increases in reactive astrocytes after spinal cord injury, inhibiting neural regrowth, while knockdown promotes regrowth [27, 28]. Developmental effects in Oasis-null mice include larger numbers of neural precursor cells (NPCs) and fewer GFAP-expressing astrocytes [26]. OASIS and other CREB3 group TFs in mammals upregulate secretory pathway genes, often in response to endoplasmic reticulum (ER) stress [29, 30, 55]. It was proposed that astrocyte-secreted proteins might be part of the response to injury or stress in secretory cells mediated by OASIS [26]. Although the above studies focus on astrocytes in mammals; in *Drosophila*, CrebA regulates dendrite development via genes in the secretory pathway [56]. Secretory gene products are required for the remodeling and extension of dendritic processes during development and following dendritic injury.

DANs are neurosecretory cells and in dopaminergic cell lines, dopamine exposure induced ER stress which phenocopied the unfolded protein response implicated in Parkinson’s disease (PD) [57]. Induction of ATF ER stress response transcription factors (TFs) is involved in PD pathophysiology, and a role for ATF6 in mitigating PD progression was observed [58]. A recent report documented strong upregulation of ATF3 in midbrain DANs following 6-OHDA insult [42]. We describe the interplay between epigenetic regulation, ATF transcription factors, ER stress, and its connection to PD in subsequent sections.

### *Hr38*/Nurr1/NR4A2

A second strong behavioral response was found upon downregulation of the fly ARG-TF *Hr38*, a homolog of the human Nurr1/NR4A2 ARG, whose continued transcription is required for maintenance of adult mammalian dopaminergic neurons (DANs) [33]. Moreover, increasing Nurr1 expression helps generate DANs from iPSCs [34], while the expression of a nuclear-targeted Nurr1 fragment in dopaminergic SH-SY5Y cells increased tyrosine hydroxylase expression and protected cells from the DAN-specific neurotoxin 6-hydroxydopamine [35]. Nurr1 expression is reduced in some cases of PD, and the authors speculate that protein expression of fragments of Nurr1 may alleviate PD neurodegeneration [35]. Furthermore, HDAC inhibitors increase dopamine aminotransferase (DAT) expression in DANs via acetylation of Nurr1 promoters and increased Nurr1 transcription [59]. Although both HAT and HDAC inhibitors and activators have been studied in PD, their roles are not completely understood [60].

**Fig. 1:**
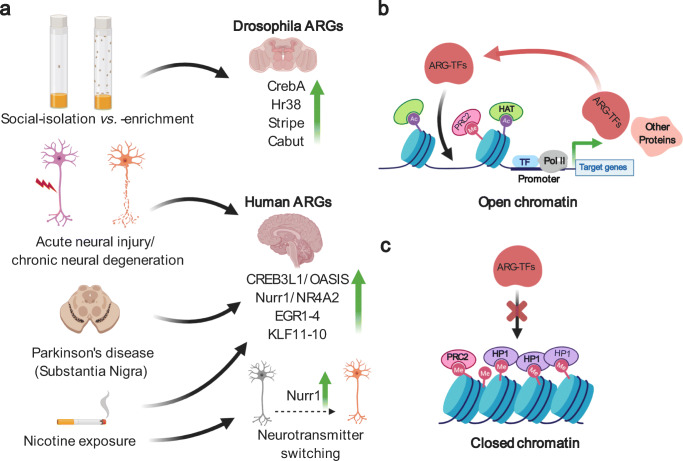
Interplay between epigenetic mechanisms and ARGs. **a** *Drosophila* ARGs and their homologs in humans. ARGs are upregulated due to both acute perturbations such as neural injury and chronic perturbation such as social isolation vs. social enrichment; Parkinson’s disease (PD); nicotine exposure; neuronal regeneration; and during neurotransmitter switching. **b** ARG-TFs are under epigenetic regulation. The top panel shows a bivalently marked nucleosome (Ac, acetylation mark; Me, methylation mark), downstream to this nucleosome TFs can interact with Pol II to continue transcription of the target gene, in this case transcribing an ARG-TF. These ARG-TFs can in turn regulate the expression of their downstream targets. **c** Repressive marks mediated by PRC1 and PRC2 can condense the chromatin and restrict access of ARG-TFs to downstream targets. Binding of PRC1 produces H3K9me3 marks, leading to HP1 recruitment and eventually gene repression

Neonatal stressors can affect Nurr1 expression in non-dopaminergic neurons. Neonatal exposure to nicotine increases Nurr1 levels in some glutamatergic VTA neurons without triggering TH expression; however, re-exposure to nicotine in adulthood increases Nurr1 expression further in these “poised” neurons, leading to a switch towards DAN phenotype [36] (Fig. 1). The subject of neurotransmitter switching, which in several cases involves dopamine and is triggered by environmental inputs, was recently reviewed [61].

#### *Cabut/*KLF10-11

The ARG *Cabut/*KLF10-11 was upregulated in DANs of socially enriched flies, and its knockdown in DANs reduced sleep phenotype modulation by social experience [5]. In mammals, KLF11/TIEG2 and MAO (monoamine oxidase) signaling is increased in the prefrontal cortex by chronic social defeat and is associated with depression and KLF11 regulates the MAO response [38, 62]. KLF11/TIEG2 regulates multiple pathways via different domains that cause different epigenetic effects on chromatin through binding with the SIN3A complex, the HP1 complex, and WD40-containing proteins [39]. In *Drosophila* DANs, we found upregulation of *Brms1*—a member of the histone deacetylase Sin3A repressor complex, upon social enrichment; its targeted knockdown in DANs produced sleep phenotypes similar to that of *Cabut/*KLF10-11 knockdown [5].

KLF11 increases DRD2 dopamine receptor expression through the HAT p300 but also limits the increase through the HP1/PRC1 complex [40]. Since DRD2 auto-receptor expression in fly DANs rescued degeneration caused by the PD selective neurotoxin MPP(+) [63], the role of KLF11/TIEG2 and epigenetic factors in PD requires further attention.

#### *sr*/EGR1/EGR2

The fourth ARG-TF we studied is called *stripe* (*sr*) in flies and has homology to EGR2 and EGR1 in humans. In rat Schwann cells, EGR2 upregulates myelination in some contexts and interacts with c-Jun [43]. EGR1, previously known as ZENK in birds [64], is well studied as an ARG involved with both short- and long-term responses to conspecific song in the brains of zebra finches [41, 64]. It was shown that in the hippocampus of mice, EGR1 directly interacts with lysine-specific demethylase 1 (LSD1), allowing a permissive chromatin state, and upon stress, this leads to increased Egr1 expression and a high-anxiety phenotype [44]. Similarly, the reduction of Egr1 levels by LSD1 knockout, which promotes repressive chromatin state, leads to a low-anxiety phenotype [44].

### Consistent ARG Response to Activation of Mammalian and Fly DANs

Above we reviewed the roles of ARGs individually. However, the question remains, whether the homologs of these ARGs in mammals respond as a group in a similar way to neural stimulation. A published study using cultured rat midbrain dopaminergic (mDA) neurons compared the transcriptional state of neurons before and after depolarization [65], analogous to the experiments performed by stimulating dopaminergic neurons in flies which identified ARGs in fly DANs [17]. Given the similarities between the rat and the fly studies, we used the GEO profiles tool on the array data (GEO accession GDS2774) from the rat mDA study [65] to see which ARG-TFs had expression correlated to that of EGR2 in rat mDA neurons. Classic ARGs such as Fos, Jun, Srf, and Arc covaried with Egr2, as did ATF2, ATF3, and ATF4 (see the section on CrebA/Oasis above), Klf10, Egr1, and Nr4a1, Nr4a2/Nurr1, and Nr4a3. Thus, activity in cultured rat mDA neurons produces a similar set of ARG changes to those observed in fruit fly DANs.

## ARGs, Epigenetic Regulation, and Parkinson’s Disorders

Epigenetics has emerged as one of the fundamental mechanisms regulating nervous system development and function. Epigenetic mechanisms play a central role in regulating the neuronal response to stress, injury, and regeneration (Fig. 1). Epigenetic disruption can cause neurological disorders such as Parkinson’s, Huntington’s, schizophrenia, autism, and addiction, many of which target the DAN network [60, 66–69]. A recent study searching for genes differentially expressed in Parkinson’s, Alzheimer’s, frontotemporal dementia, and amyotrophic lateral sclerosis found three major hub genes, including EGFR, CDC42, and CREBBP. CREBBP is a histone acetylase that partners with CREB to increase histone acetylation and gene expression at CREB-binding sites [70]. Another intersectional study of genes differentially expressed in Parkinson’s and Huntington’s diseases implicated targets of CREB signaling [71]. The authors noted that shared pathways “suggest that biological processes related to neuronal plasticity are active in both of these diseases and may even indicate that neuroprotective or neuro-regenerative processes are a component of the neurodegenerative response” which we find interesting in light of our results on transcriptional and epigenetic plasticity in the DAN network in response to social isolation stress. Together, it suggests that prolonged environmental stress, similar to neurodegenerative disorders, might promote a metastable epigenetic state in neurons using common transcriptional and epigenetic mechanisms.

In neural degeneration and regeneration, the roles of epigenetic mechanisms are under intense investigation. Studies utilizing histone acetylases (HATs), histone deacetylases (HDAC), and their inhibitors (HDACi) suggest a diverse and context-specific role of histone acetylation; e.g., HDAC1 but not HDAC3 was shown to play a prominent role in initiating axon regeneration after injury [72]. In late-stage Parkinson’s, cells in the substantia nigra pars compacta (SNc) show overall increases in histone H3 acetylation, but this was demonstrated to be due to a mix of H3 hypo-acetylation in DANs and H3 hyper-acetylation in activated microglia [73]. A recent study showed that HDAC2 mRNA and protein levels were higher in SNc microglia from PD than in controls [74]. Human telencephalic/mesencephalic microglial lines increased HDAC2 expression upon bacterial liposaccharide treatment, suggesting that the deacetylation of genes in microglia is part of a response to inflammation in PD [74]. Treatment with an inhibitor of the histone deacetylase SIRT2 (AGK2) resulted in neuroprotection of DANs in a PD model [73]. Nuclear localization of histone deacetylase HDAC4 in DANs reduced CREB signaling and increased DAN loss. CREB repression was also seen in a phosphorylation-deficient mutant of HDAC4, which causes it to translocate to the nucleus, activating cell death in PC12 rat cells expressing A53T-synuclein mutation [31]. It must be noted that different HDACs may take on opposing roles. These data suggest that targeting of specific HDACi’s to SNc microglia may be a more selective mode of therapy with fewer side effects. It is important to note that various FDA-approved HDACi drugs for cancer treatment have gained prominence in the field of neuropsychiatry due to their known effects in animal models, including suberoylanilide hydroxamic acid (SAHA, aka Vorinostat) and romidepsin (aka Istodax) [60, 75].

The well-studied α-synuclein (SNCA) protein and its aggregates play an important role in several neurodegenerative disorders, including PD illustrating the complexity of epigenetic regulation in this disease [76]. α-Synuclein has been associated with several kinds of epigenetic marks in SNc DANs, including reduced DNA methylation in intron 1 of the *SNCA* gene [77, 78], and reduced histone H3 acetylation [73, 79, 80]. Furthermore, histone acetylase p300 increases α-synuclein aggregation, which might in turn reduce histone acetylation [81]. A single-nucleotide polymorphism in an enhancer of *SNCA* reduces the binding of the repressive complex EMX2/NKX6-1 [82]. EMX2/NKX6-1 recruits HDAC1, so limiting EMX2/NKX6 binding increases histone acetylation and thus may increase SNCA expression. In this context, HDAC inhibitors may worsen PD symptoms, as shown for the class 1 (HDAC 1 and 2) HDACi valproic acid (VPA), which can induce Parkinsonism in the elderly [83]. This counterexample to the therapeutic use of HDACi’s in PD emphasizes the point mentioned above that targeting of specific HDACi’s and agonists to specific tissue types may be needed to achieve the full potential of epigenetic therapy in PD.

Histone side chains are also marked by methylation, which is longer lasting than acetylation. H3K4me3 is considered a mark for gene activation, whereas H3K9me3 and H3K27me3 are considered marks for gene repression. The latter are usually created by members of the polycomb repressive complexes (PRC), with PRC1 yielding H3K9me3 and PRC2 producing H3K27me3 marks. PRC modification of chromatin is one of the main mechanisms of transcriptional repression, both during development and normal function, described in a comprehensive set of reviews [84]. Modifiers of histone methyl marks change ARG transcriptional states and are involved in tuning the response of the nervous system to psychosocial stress [41, 44]. H3K27me3 PRC2 marks are often associated with promoters of genes activated after neural injury, and reduction of polycomb marks activates a subset of genes in the injury program [85]. During development, so-called bivalent promoters have a mix of activating H3K4me3 and repressive PRC marks, but it has been suggested that even in mature neurons bivalent promoters persist and that mis-marked bivalents may contribute to Huntington’s disease [86]. We find this suggestion intriguing since we found that shifts in the balance between these three histone methyl marks were prominent in fly DANs due to social deprivation vs. social enrichment. Gene ontology (GO) functional analysis using DAVID bioinformatics analysis tool [87] suggested that social enrichment increased levels of PRC2 marks in DAN functional gene groups such as ion channels and neuropeptide signaling at the expense of PRC1 and H3K4me3 marks, while genes involved in pathways of neural regulation such as MAPK and WNT signaling etc. had contrasting PRC2 mark declines and PRC1 mark increases [5]. As methyl and acetyl marks on the same histone lysine residue have competing roles, our observation that social enrichment invokes a broad program of PRC1 and PRC2 methyl mark shifts that are specific to certain functional gene types may have relevance to the more extreme changes in SNc DAN’s found in PD and other neurodegenerative disorders (Fig. 1).

Indeed, knockdown of PRC2 histone methyltransferase genes in striatal medium spiny neurons (MSNs) caused a progressive neural degeneration syndrome by allowing the de-repression of cell death and non-MSN genes [88]. Notably, among PRC2 target genes in MSNs, over 40% had H3K4me3/H3K27me3 patterns typical of genes with bivalent promoters [88]. Prolonged treatment with L-DOPA in PD patients is known to cause dyskinesia, a movement disorder [89]. A study of MSNs targets in the striatum of dopamine signaling found that prolonged administration of L-DOPA caused phosphorylation of a serine adjacent to H3K27me3, displacement of PRC2 from such sites, and de-repression of PRC2 target genes [37]. Among these genes were NR2A4 and EGR2, two of the ARGs that we found responded strongly in DANs to social stimulation in *Drosophila* (Table 1). Another study that used cell type-specific mRNA expression profiling upon L-DOPA treatment also found induction of the ARGs NR2A4 (Nurr1) and CREB in striatal spiny projection neurons [32]. Södersten et al. suggest that NR2A4 and EGR2 may be bivalently regulated by PRC2 marks in adult neurons, which if true would suggest an interplay between transcriptional upregulation of ARGs and epigenetic changes in PD [37].

Following up on these results, a recent study measured histone marks and gene expression in midbrain DANs with or without exposure to neurotoxic 6-OHDA or methamphetamine [42]. Bivalently marked H3K4me3/H3K27me3 genes were significantly enriched among genes upregulated by neurotoxic stress, suggesting that bivalency in promoters of genes in adult neurons is predominantly a sign of normal-state repression. One such gene was ATF3, a member of the ER stress-responsive ATF group, while another was FOXA1, which is involved, like Nurr1/NR4A2, in the maintenance of adult DANs [90]. During DAN development, Foxa1 is a coactivator of Nurr1/NR2A4, and together, these two studies suggest that three TFs essential for adult DAN maintenance (EGR2, NR2A4, and Foxa1) and one involved in ER stress response (ATF3) are bivalently marked with H3K4me3/H3K27me3 in spite of measurable and continued expression in adult DANs [37, 42]. As a statistical check of this suggestion, we used the gene ontology (GO) tool GOrilla [91], to see what gene groups are overrepresented among genes with bivalent promoter H3K4me3/H3K27me3 marks identified by [42]. Significant GO groups included associative learning, neuropeptide signaling, ion channel activity, and transcriptional regulation—each of which was also significantly enriched in our fly DAN gene cluster with the strongest H3K4me3 and H3K27me3 changes in response to social enrichment ((Fig. 3, cluster 8) of [5]). These results from mouse, rat, and fruit fly DANs strongly suggest that activity and/or stress in mature dopaminergic neurons have shared transcriptional responses mediated by ARG-TFs and that some genes involved in these processes have unusual bivalent promoter epigenetic marks. We suggest that as therapeutic investigations of HDAC, HAT, and histone methylase/demethylase compounds continues, careful attention should be paid to the effect of such treatments on PRC2-associated bivalent promoters.

In our study, many neural signaling genes showed a decrease in H3K27me3 and an increase in mRNA expression in socially deprived fly DANs, compared with those in socially enriched groups. In flies, social isolation produces other behavioral changes associated with stress, such as increased aggressiveness, locomotion, and decreased sleep [5]. Taken together, results from organisms as different as flies, mice, and humans suggest that DANs and some of their target neurons and supporting glia have epigenetic and transcriptional programs responsive to minor (social) or major (neurotoxic) stresses. These programs differ by cell type but share some similarities in the common roles of ARGs, epigenetic marks, and in some cases bivalent promoters even in the adult, fully differentiated state.

### Achieving Cell Type Specificity from Broad Expression of ARGs

Given that ARGs are broadly expressed in the nervous system in response to stimulus [17, 92], it will be important to understand how they can induce cell type-specific gene expression programs in order to achieve effective therapeutic interventions in neurodegenerative disorders. Similar to the ARGs, their interacting epigenetic factors, mentioned throughout this review, are also broadly expressed. Several studies have identified coordinated response by ARGs in the nervous system inducing a global response from the genome and epigenome, reviewed in [41, 93].

**Fig. 2:**
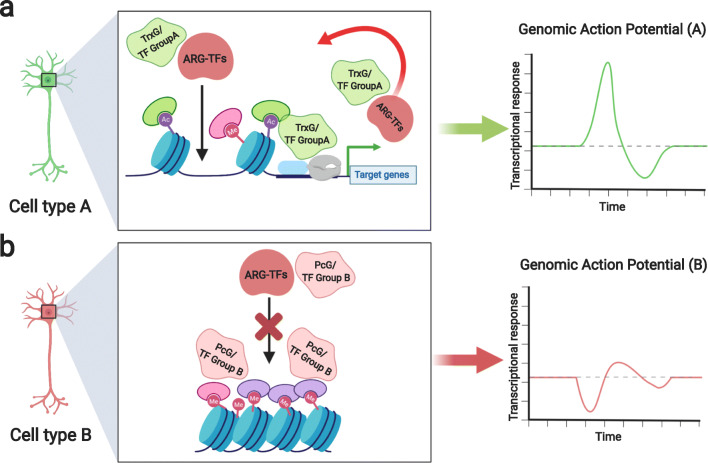
Distinct cell-type-specific outcomes by broadly expressed ARGs. Distinct sets of pre-existing cell type-specific transcription factors plus the trithorax group (TrxG) and polycomb group (PcG) proteins provide different transcriptional platforms due to which broadly expressed ARGs can produce distinct genomic responses. Examples showing **a** activating transcription factors (group A) along with TrxG proteins give rise to a genomic action potential (gAP) characterized by overall increased transcription. **b** Repressive transcription factors (group B) along with PcG proteins give rise to a gAP characterized by overall reduced transcription

It is likely that broadly expressed ARG-TFs interact with platforms of TF and histone modifiers distinct across various cell types, leading to the induction of distinct subsets of target genes. This is reminiscent of how broadly expressed master regulators of animal development such as Hox TFs can specify pattern of individual body segments by interacting with pre-existing transcriptional and epigenetic platforms laid down by pioneer factors including polycomb and trithorax group proteins [94–96]. We therefore propose that ARG-TFs utilize a mechanism similar to those employed by broadly expressed Hox genes to achieve cell-type specificity by interacting with pre-existing pioneer factors including epigenetic machinery and transcriptional regulators (Fig. 2). These interactions can aid ARG-TFs in transducing a global cell-type-specific response, named a “genomic action potential,” or gAP [41].

Therefore, ARGs and epigenetic histone modifications are associated with both normal development and function in DANs and other neurons, and with switches in cell types such as neurotransmitter switching [61]. Our findings show that socially induced DAN state changes are accompanied by pervasive epigenetic changes, which may be related to the balance between stimulatory and repressive PRC marks. Such balanced epigenetic regulation in adult neurons is very similar to the bivalent promoters involved in neurodevelopment and found in adult neurons at promoters of ARGs [41]. If significant subsets of repair pathway genes are regulated by ARGs plus epigenetic switching, further study of these mechanisms may contribute to improved regeneration therapy for Parkinson’s disease and other neurodegenerative disorders.

### Future Directions

How neuronal stimulation can lead to coordinated responses in gene expression and how specificity is achieved in these responses across various neural circuits is a long-standing question. We hypothesize that broadly expressed ARGs might interact with pre-existing cell-type-specific pioneering factors to impart specificity. It would therefore be important to identify these TFs across various cell types and how they interact with ARGs and regulate downstream targets. Central roles of these ARGs in several neuropsychiatric disorders, as discussed throughout this review, make them attractive candidates for therapeutic intervention. Recent technological advancements in single-cell transcriptional profiling to identify changes in ARGs and transcriptional states [97], coupled with single-cell ChIP-seq technologies [98, 99] will advance mechanistic understanding of how the neuronal epigenome responds to environmental stimuli via ARGs and affects behavior. Such efforts need to be aided by mechanistic understanding gained from future studies as well as recent advances in epigenetic engineering [100–103]. These approaches restrict epigenetic changes to specific loci on the genome without affecting chromatin state globally, which can be particularly useful in developing targeted therapies involving epigenetic alterations for neurodegenerative disorders.

## References

[1] Vieira MS, Goulart VAM, Parreira RC, Oliveira-Lima OC, Glaser T, Naaldijk YM, Ferrer A, Savanur VH, Reyes PA, Sandiford O, Rameshwar P, Ulrich H, Pinto MCX, Resende RR (2018). Decoding epigenetic cell signaling in neuronal differentiation. Semin Cell Dev Biol.

[2] Weaver ICG, Cervoni N, Champagne FA, D’Alessio AC, Sharma S, Seckl JR, Dymov S, Szyf M, Meaney MJ (2004). Epigenetic programming by maternal behavior. Nat Neurosci.

[3] Niwa M, Jaaro-Peled H, Tankou S, Seshadri S, Hikida T, Matsumoto Y, Cascella NG, Kano SI, Ozaki N, Nabeshima T, Sawa A (2013). Adolescent stress–induced epigenetic control of dopaminergic neurons via glucocorticoids. Science.

[4] Pusalkar M, Suri D, Kelkar A, Bhattacharya A, Galande S, Vaidya VA (2015). Early stress evokes dysregulation of histone modifiers in the medial prefrontal cortex across the life span. Dev Psychobiol.

[5] Agrawal P, Chung P, Heberlein U, Kent CF (2019). Enabling cell-type-specific behavioral epigenetics in Drosophila: a modified high-yield INTACT method reveals the impact of social environment on the epigenetic landscape in dopaminergic neurons. BMC Biol.

[6] Ohira K (2020). Dopamine as a growth differentiation factor in the mammalian brain. Neural Regen Res.

[7] Furuyashiki T, Kitaoka S (2019). Neural mechanisms underlying adaptive and maladaptive consequences of stress: roles of dopaminergic and inflammatory responses. Psychiatry Clin Neurosci.

[8] Stanwood GD (2019). Dopamine and stress. Stress Physiol Biochem Pathol.

[9] Asahina K, Watanabe K, Duistermars BJ, Hoopfer E, González CR, Eyjólfsdóttir EA, Perona P, Anderson DJ (2014). Tachykinin-expressing neurons control male-specific aggressive arousal in Drosophila. Cell.

[10] Agrawal P, Kao D, Chung P, Looger LL (2020) The neuropeptide Drosulfakinin regulates social isolation-induced aggression in Drosophila. J Exp Biol 223:jeb207407. 10.1242/jeb.207407 10.1242/jeb.207407 PMC7033730 31900346

[11] Asahina K (2017). Neuromodulation and strategic action choice in *Drosophila* aggression. Annu Rev Neurosci.

[12] Hoopfer ED (2016). Neural control of aggression in Drosophila. Curr Opin Neurobiol.

[13] Ganguly-Fitzgerald I, Donlea J, Shaw PJ (2006). Waking experience affects sleep need in Drosophila. Science.

[14] Lone SR, Potdar S, Srivastava M, Sharma VK (2016). Social experience is sufficient to modulate sleep need of drosophila without increasing wakefulness. PLoS One.

[15] Kim Y-K, Saver M, Simon J, Kent CF, Shao L, Eddison M, Agrawal P, Texada M, Truman JW, Heberlein U (2018). Repetitive aggressive encounters generate a long-lasting internal state in Drosophila melanogaster males. Proc Natl Acad Sci.

[16] Alekseyenko OV, Chan Y-B, Li R, Kravitz EA (2013). Single dopaminergic neurons that modulate aggression in Drosophila. Proc Natl Acad Sci U S A.

[17] Chen X, Rahman R, Guo F, Rosbash M (2016). Genome-wide identification of neuronal activity-regulated genes in Drosophila. Elife.

[18] Greenberg ME, Ziff EB, Greene LA (1986). Stimulation of neuronal acetylcholine receptors induces rapid gene transcription. Science.

[19] Yap EL, Greenberg ME (2018). Activity-regulated transcription: bridging the gap between neural activity and behavior. Neuron.

[20] Sheng M, Greenberg ME (1990). The regulation and function of c-fos and other immediate early genes in the nervous system. Neuron.

[21] Fujita N, Nagata Y, Nishiuchi T, Sato M, Iwami M, Kiya T (2013). Visualization of neural activity in insect brains using a conserved immediate early gene, Hr38. Curr Biol.

[22] Sommerlandt FMJ, Brockmann A, Rössler W, Spaethe J (2019). Immediate early genes in social insects: a tool to identify brain regions involved in complex behaviors and molecular processes underlying neuroplasticity. Cell Mol Life Sci.

[23] Shin JE, Cho Y (2017). Epigenetic regulation of axon regeneration after neural injury. Mol Cells.

[24] Wallace DL, Han MH, Graham DL, Green TA, Vialou V, Iñiguez SD, Cao JL, Kirk A, Chakravarty S, Kumar A, Krishnan V, Neve RL, Cooper DC, Bolaños CA, Barrot M, McClung CA, Nestler EJ (2009). CREB regulation of nucleus accumbens excitability mediates social isolation-induced behavioral deficits. Nat Neurosci.

[25] Honma Y, Kanazawa K, Mori T, Tanno Y, Tojo M, Kiyosawa H, Takeda J, Nikaido T, Tsukamoto T, Yokoya S, Wanaka A (1999). Identification of a novel gene, OASIS, which encodes for a putative CREB/ATF family transcription factor in the long-term cultured astrocytes and gliotic tissue. Mol Brain Res.

[26] Saito A (2014). Physiological functions of endoplasmic reticulum stress transducer OASIS in central nervous system. Anat Sci Int.

[27] Iseki K, Hagino S, Nikaido T, Zhang Y, Mori T, Yokoya S, Hozumi Y, Goto K, Wanaka A, Tase C (2012). Gliosis-specific transcription factor OASIS coincides with proteoglycan core protein genes in the glial scar and inhibits neurite outgrowth. Biomed Res.

[28] Sumida Y, Kamei N, Suga N, Ochi M, Adachi N (2018). The endoplasmic reticulum stress transducer old astrocyte specifically induced substance positively regulates glial scar formation in spinal cord injury. Neuroreport.

[29] Fox RM, Hanlon CD, Andrew DJ (2010). The CrebA/Creb3-like transcription factors are major and direct regulators of secretory capacity. J Cell Biol.

[30] Greenwood M, Greenwood MP, Paton JFR, Murphy D (2015). Transcription factor CREB3L1 regulates endoplasmic reticulum stress response genes in the osmotically challenged rat hypothalamus. PLoS One.

[31] Wu Q, Yang X, Zhang L, Zhang Y, Feng L (2017). Nuclear accumulation of histone deacetylase 4 (HDAC4) exerts neurotoxicity in models of Parkinson’s disease. Mol Neurobiol.

[32] Heiman M, Heilbut A, Francardo V, Kulicke R, Fenster RJ, Kolaczyk ED, Mesirov JP, Surmeier DJ, Cenci MA, Greengard P (2014). Molecular adaptations of striatal spiny projection neurons during levodopa-induced dyskinesia. Proc Natl Acad Sci U S A.

[33] Kadkhodaei B, Ito T, Joodmardi E, Mattsson B, Rouillard C, Carta M, Muramatsu SI, Sumi-Ichinose C, Nomura T, Metzger D, Chambon P, Lindqvist E, Larsson NG, Olson L, Bjorklund A, Ichinose H, Perlmann T (2009). Nurr1 is required for maintenance of maturing and adult midbrain dopamine neurons. J Neurosci.

[34] Salemi S, Baktash P, Rajaei B, Noori M, Amini H, Shamsara M, Massumi M (2016). Efficient generation of dopaminergic-like neurons by overexpression of Nurr1 and Pitx3 in mouse induced pluripotent stem cells. Neurosci Lett.

[35] Paliga D, Raudzus F, Leppla SH, Heumann R, Neumann S (2019). Lethal factor domain-mediated delivery of Nurr1 transcription factor enhances tyrosine hydroxylase activity and protects from neurotoxin-induced degeneration of dopaminergic cells. Mol Neurobiol.

[36] Romoli B, Lozada AF, Sandoval IM, Manfredsson FP, Hnasko TS, Berg DK, Dulcis D (2019). Neonatal nicotine exposure primes midbrain neurons to a dopaminergic phenotype and increases adult drug consumption. Biol Psychiatry.

[37] Södersten E, Feyder M, Lerdrup M, Gomes AL, Kryh H, Spigolon G, Caboche J, Fisone G, Hansen K (2014). Dopamine signaling leads to loss of polycomb repression and aberrant gene activation in experimental parkinsonism. PLoS Genet.

[38] Harris S, Johnson S, Duncan JW, Udemgba C, Meyer JH, Albert PR, Lomberk G, Urrutia R, Ou XM, Stockmeier CA, Wang JM (2015). Evidence revealing deregulation of the KLF11-Mao a pathway in association with chronic stress and depressive disorders. Neuropsychopharmacology.

[39] Calvo E, Grzenda A, Lomberk G, Mathison A, Iovanna J, Urrutia R (2014). Single and combinatorial chromatin coupling events underlies the function of transcript factor krüppel-like factor 11 in the regulation of gene networks. BMC Mol Biol.

[40] Seo S, Lomberk G, Mathison A, Buttar N, Podratz J, Calvo E, Iovanna J, Brimijoin S, Windebank A, Urrutia R (2012). Krüppel-like factor 11 differentially couples to histone acetyltransferase and histone methyltransferase chromatin remodeling pathways to transcriptionally regulate dopamine D2 receptor in neuronal cells. J Biol Chem.

[41] Clayton DF, Anreiter I, Aristizabal M, Frankland PW, Binder EB, Citri A (2019). The role of the genome in experience-dependent plasticity: extending the analogy of the genomic action potential. Proc Natl Acad Sci.

[42] Södersten E, Toskas K, Rraklli V, Tiklova K, Björklund ÅK, Ringnér M, Perlmann T, Holmberg J (2018). A comprehensive map coupling histone modifications with gene regulation in adult dopaminergic and serotonergic neurons. Nat Commun.

[43] Tammia M, Mi R, Sluch VM, Zhu A, Chung T, Shinn D, Zack DJ, Höke A, Mao HQ (2018). Egr2 overexpression in Schwann cells increases myelination frequency in vitro. Heliyon.

[44] Rusconi F, Grillo B, Ponzoni L, Bassani S, Toffolo E, Paganini L, Mallei A, Braida D, Passafaro M, Popoli M, Sala M, Battaglioli E (2016). LSD1 modulates stress-evoked transcription of immediate early genes and emotional behavior. Proc Natl Acad Sci.

[45] Jenkins R, O’shea R, Thomas KL, Hunt SP (1993). c-jun Expression in substantia nigra neurons following striatal 6-hydroxydopamine lesions in the rat. Neuroscience.

[46] Jenkins R, Tetzlaff W, Hunt SP (1993). Differential expression of immediate early genes in rubrospinal neurons following axotomy in rat. Eur J Neurosci.

[47] Arthur-Farraj PJ, Latouche M, Wilton DK, Quintes S, Chabrol E, Banerjee A, Woodhoo A, Jenkins B, Rahman M, Turmaine M, Wicher GK, Mitter R, Greensmith L, Behrens A, Raivich G, Mirsky R, Jessen KR (2012). c-Jun reprograms Schwann cells of injured nerves to generate a repair cell essential for regeneration. Neuron.

[48] Friggi-Grelin F, Coulom H, Meller M (2003). Targeted gene expression in Drosophila dopaminergic cells using regulatory sequences from tyrosine hydroxylase. J Neurobiol.

[49] Nakagawa S, Kim JE, Lee R, Chen J, Fujioka T, Malberg J, Tsuji S, Duman RS (2002). Localization of phosphorylated cAMP response element-binding protein in immature neurons of adult hippocampus. J Neurosci.

[50] Landeira BS, Santana TTDS, Araújo JADM (2018). Activity-independent effects of CREB on neuronal survival and differentiation during mouse cerebral cortex development. Cereb Cortex.

[51] Lee J-K, Chung J, Druey KM, Tansey MG (2012). RGS10 exerts a neuroprotective role through the PKA/c-AMP response-element (CREB) pathway in dopaminergic neuron-like cells. J Neurochem.

[52] Feldmann KG, Chowdhury A, Becker JL, McAlpin N’G, Ahmed T, Haider S, Richard Xia JX, Diaz K, Mehta MG, Mano I (2019). Non-canonical activation of CREB mediates neuroprotection in a Caenorhabditis elegans model of excitotoxic necrosis. J Neurochem.

[53] Chalovich EM, Zhu JH, Caltagarone J, Bowser R, Chu CT (2006). Functional repression of cAMP response element in 6-hydroxydopamine-treated neuronal cells. J Biol Chem.

[54] Kreutzmann JC, Havekes R, Abel T, Meerlo P (2015). Sleep deprivation and hippocampal vulnerability: changes in neuronal plasticity, neurogenesis and cognitive function. Neuroscience.

[55] Barbosa S, Fasanella G, Carreira S, Llarena M, Fox R, Barreca C, Andrew D, O’Hare P (2013). An orchestrated program regulating secretory pathway genes and cargos by the transmembrane transcription factor CREB-H. Traffic.

[56] Iyer SC, Ramachandran Iyer EP, Meduri R, Rubaharan M, Kuntimaddi A, Karamsetty M, Cox DN (2013). Cut, via CrebA, transcriptionally regulates the COPII secretory pathway to direct dendrite development in Drosophila. J Cell Sci.

[57] Dukes AA, Van Laar VS, Cascio M, Hastings TG (2008). Changes in endoplasmic reticulum stress proteins and aldolase A in cells exposed to dopamine. J Neurochem.

[58] Egawa N, Yamamoto K, Inoue H, Hikawa R, Nishi K, Mori K, Takahashi R (2011). The endoplasmic reticulum stress sensor, ATF6α, protects against neurotoxin-induced dopaminergic neuronal death. J Biol Chem.

[59] Green AL, Zhan L, Eid A, Zarbl H, Guo GL, Richardson JR (2017). Valproate increases dopamine transporter expression through histone acetylation and enhanced promoter binding of Nurr1. Neuropharmacology.

[60] Hegarty SV, Sullivan AM, O’Keeffe GW (2016). The epigenome as a therapeutic target for Parkinson’s disease. Neural Regen Res.

[61] Spitzer NC (2017). Neurotransmitter switching in the developing and adult brain. Annu Rev Neurosci.

[62] Grunewald M, Johnson S, Lu D, Wang Z, Lomberk G, Albert PR, Stockmeier CA, Meyer JH, Urrutia R, Miczek KA, Austin MC, Wang J, Paul IA, Woolverton WL, Seo S, Sittman DB, Ou XM (2012). Mechanistic role for a novel glucocorticoid-KLF11 (TIEG2) protein pathway in stress-induced monoamine oxidase A expression. J Biol Chem.

[63] Wiemerslage L, Schultz BJ, Ganguly A, Lee D (2013). Selective degeneration of dopaminergic neurons by MPP+ and its rescue by D2 autoreceptors in Drosophila primary culture. J Neurochem.

[64] Jarvis ED, Nottebohm F (1997). Motor-driven gene expression. Proc Natl Acad Sci U S A.

[65] Volpicelli F, Caiazzo M, Greco D, Consales C, Leone L, Perrone-Capano C, D’Amato LC, Porzio U (2007). Bdnf gene is a downstream target of Nurr1 transcription factor in rat midbrain neurons in vitro. J Neurochem.

[66] Renthal W, Kumar A, Xiao G, Wilkinson M, Covington HE, Maze I, Sikder D, Robison AJ, LaPlant Q, Dietz DM, Russo SJ, Vialou V, Chakravarty S, Kodadek TJ, Stack A, Kabbaj M, Nestler EJ (2009). Genome-wide analysis of chromatin regulation by cocaine reveals a role for sirtuins. Neuron.

[67] Walker DM, Cates HM, Heller EA, Nestler EJ (2015). Regulation of chromatin states by drugs of abuse. Curr Opin Neurobiol.

[68] Akbarian S (2010). The molecular pathology of schizophrenia--focus on histone and DNA modifications. Brain Res Bull.

[69] Francelle L, Lotz C, Outeiro T, Brouillet E, Merienne K (2017). Contribution of Neuroepigenetics to Huntington’s disease. Front Hum Neurosci.

[70] Habib R, Noureen N, Nadeem N (2018). Decoding common features of neurodegenerative disorders: from differentially expressed genes to pathways. Curr Genomics.

[71] Labadorf A, Choi SH, Myers RH (2018) Evidence for a pan-neurodegenerative disease response in Huntington’s and Parkinson’s disease expression profiles. Front Mol Neurosci 10. 10.3389/fnmol.2017.00430 10.3389/fnmol.2017.00430 PMC5768647 29375298

[72] Chen J, Shifman M (2016). The expression of histone deacetylases and the regenerative abilities of spinal-projecting neurons after injury. Neural Regen Res.

[73] Harrison IF, Smith AD, Dexter DT (2018). Pathological histone acetylation in Parkinson’s disease: neuroprotection and inhibition of microglial activation through SIRT 2 inhibition. Neurosci Lett.

[74] Tan Y, Delvaux E, Nolz J, Coleman PD, Chen S, Mastroeni D (2018). Upregulation of histone deacetylase 2 in laser capture nigral microglia in Parkinson’s disease. Neurobiol Aging.

[75] Sharma S, Taliyan R (2015). Targeting histone deacetylases: a novel approach in Parkinson’s disease. Park Dis.

[76] Paumier KL, Luk KC, Manfredsson FP, Kanaan NM, Lipton JW, Collier TJ, Steece-Collier K, Kemp CJ, Celano S, Schulz E, Sandoval IM, Fleming S, Dirr E, Polinski NK, Trojanowski JQ, Lee VM, Sortwell CE (2015). Intrastriatal injection of pre-formed mouse α-synuclein fibrils into rats triggers α-synuclein pathology and bilateral nigrostriatal degeneration. Neurobiol Dis Dis.

[77] Jowaed A, Schmitt I, Kaut O, Wüllner U (2010). Methylation regulates alpha-synuclein expression and is decreased in Parkinson’s disease patients’ brains. J Neurosci.

[78] Matsumoto L, Takuma H, Tamaoka A, Kurisaki H, Date H, Tsuji S, Iwata A (2010). CpG demethylation enhances alpha-synuclein expression and affects the pathogenesis of Parkinson’s disease. PLoS One.

[79] Goers J, Manning-Bog AB, McCormack AL (2003). Nuclear localization of α-synuclein and its interaction with histones. Biochemistry.

[80] Kontopoulos E, Parvin JD, Feany MB (2006). Α-Synuclein acts in the nucleus to inhibit histone acetylation and promote neurotoxicity. Hum Mol Genet.

[81] Kirilyuk A, Shimoji M, Catania J, Sahu G, Pattabiraman N, Giordano A, Albanese C, Mocchetti I, Toretsky JA, Uversky VN, Avantaggiati ML (2012). An intrinsically disordered region of the acetyltransferase p300 with similarity to prion-like domains plays a role in aggregation. PLoS One.

[82] Soldner F, Stelzer Y, Shivalila CS, Abraham BJ, Latourelle JC, Barrasa MI, Goldmann J, Myers RH, Young RA, Jaenisch R (2016). Parkinson-associated risk variant in distal enhancer of α-synuclein modulates target gene expression. Nature.

[83] Mahmoud F, Tampi RR (2011) Valproic acid–induced parkinsonism in the elderly: a comprehensive review of the literature. Am J Geriatr Pharmacother 9:405–412. 10.1016/j.amjopharm.2011.09.002 10.1016/j.amjopharm.2011.09.002 21993183

[84] Pirrotta V (2017) Chapter 1 - Introduction to polycomb group mechanisms. In: Pirrotta VBT-PGP (ed) Polycomb Gr. Proteins. Academic Press, pp. 1–3. 10.1016/B978-0-12-809737-3.00001-5

[85] Ma KH, Hung HA, Srinivasan R, Xie H, Orkin SH, Svaren J (2015). Regulation of peripheral nerve myelin maintenance by gene repression through polycomb repressive complex 2. J Neurosci.

[86] Zsindely N, Bodai L (2018). Histone methylation in Huntington’s disease: are bivalent promoters the critical targets?. Neural Regen Res.

[87] Huang DW, Sherman BT, Lempicki RA (2009). Systematic and integrative analysis of large gene lists using DAVID bioinformatics resources. Nat Protoc.

[88] Von Schimmelmann M, Feinberg PA, Sullivan JM (2016). Polycomb repressive complex 2 (PRC2) silences genes responsible for neurodegeneration. Nat Neurosci.

[89] Nutt JG (1990). Levodopa-induced dyskinesia: review, observations, and speculations. Neurology.

[90] Domanskyi A, Alter H, Vogt MA, Gass P, Vinnikov IA (2014). Transcription factors Foxa1 and Foxa2 are required for adult dopamine neurons maintenance. Front Cell Neurosci.

[91] Eden E, Navon R, Steinfeld I, Lipson D, Yakhini Z (2009). GOrilla: a tool for discovery and visualization of enriched GO terms in ranked gene lists. BMC Bioinformatics.

[92] Croset V, Treiber CD, Waddell S (2018). Cellular diversity in the Drosophila midbrain revealed by single-cell transcriptomics. Elife.

[93] Gray JM, Spiegel I (2019). Cell-type-specific programs for activity-regulated gene expression. Curr Opin Neurobiol.

[94] Agrawal P, Habib F, Yelagandula R, Shashidhara LS (2011) Genome-level identification of targets of Hox protein Ultrabithorax in Drosophila: novel mechanisms for target selection. Sci Rep 1. 10.1038/srep00205 10.1038/srep00205 PMC3244697 22355720

[95] Agrawal P, Shashidhara LS (2014). ChIP for Hox proteins from Drosophila imaginal discs. Methods Mol Biol.

[96] Domsch K, Carnesecchi J, Disela V, Friedrich J, Trost N, Ermakova O, Polychronidou M, Lohmann I (2019). The Hox transcription factor Ubx stabilizes lineage commitment by suppressing cellular plasticity in Drosophila. Elife.

[97] Hrvatin S, Hochbaum DR, Nagy MA, Cicconet M, Robertson K, Cheadle L, Zilionis R, Ratner A, Borges-Monroy R, Klein AM, Sabatini BL, Greenberg ME (2018). Single-cell analysis of experience-dependent transcriptomic states in the mouse visual cortex. Nat Neurosci.

[98] Grosselin K, Durand A, Marsolier J, Poitou A, Marangoni E, Nemati F, Dahmani A, Lameiras S, Reyal F, Frenoy O, Pousse Y, Reichen M, Woolfe A, Brenan C, Griffiths AD, Vallot C, Gérard A (2019). High-throughput single-cell ChIP-seq identifies heterogeneity of chromatin states in breast cancer. Nat Genet.

[99] Rotem A, Ram O, Shoresh N, Sperling RA, Goren A, Weitz DA, Bernstein BE (2015). Single-cell ChIP-seq reveals cell subpopulations defined by chromatin state. Nat Biotechnol.

[100] Heller EA, Cates HM, Peña CJ (2014). Locus-specific epigenetic remodeling controls addiction- and depression-related behaviors. Nat Neurosci.

[101] Bashtrykov P, Jeltsch A (2017) Epigenome editing in the brain. In: Delgado-Morales R (ed) Neuroepigenomics Aging Dis Adv Exp Med Biol pp 409–424 10.1007/978-3-319-53889-1_21 28523558

[102] Vojta A, Dobrinic P, Tadic V (2016). Repurposing the CRISPR-Cas9 system for targeted DNA methylation. Nucleic Acids Res.

[103] Köeferle A, Stricker SH, Beck S (2015). Brave new epigenomes: the dawn of epigenetic engineering. Genome Med.

